# Management of Gaucher Disease Type 1 in a Resource‐Limited Setting: A Pediatric Case Study

**DOI:** 10.1002/ccr3.71100

**Published:** 2025-10-04

**Authors:** Bipesh Kumar Shah, Diwakar Koirala, Bivek Mishra, Ramesh Sapkota, Manjil Basnet

**Affiliations:** ^1^ BPKIHS Dharan Nepal

## Abstract

This case report depicts the management of an 8‐year‐old male with Gaucher Disease Type 1, manifesting as massive splenomegaly, anemia, and skeletal involvement in a resource‐constrained environment. Treated with splenectomy due to the absence of enzyme replacement therapy, it underscores the necessity for enhanced therapeutic access and comprehensive care.

## 1 Introduction

Gaucher disease (GD) constitutes a lysosomal storage disorder characterized by autosomal recessive inheritance. Predominantly, the condition stems from biallelic pathogenic variants within the GBA1 gene, precipitating a deficiency in β‐glucocerebrosidase (GCase) activity. In rarer instances, GD manifests due to mutations in the PSAP gene, responsible for encoding saposin C. The resultant enzymatic insufficiency engenders an accumulation of glucocerebroside substrate within various tissues, culminating in the generation of Gaucher cells, which impair the normative functionality of specific visceral organs. Clinically, GD is stratified into three distinct phenotypes based on the absence (type I) or presence and extent (types II and III) of central nervous system (CNS) involvement. GD is typified by a constellation of clinical features, including thrombocytopenia, anemia, hepatosplenomegaly, and skeletal abnormalities. Nonetheless, individuals harboring the disease‐causing genetic variants typically remain asymptomatic. Consequently, the clinical trajectory of GD may diverge significantly between heterozygous carriers and those overtly affected by the condition [1].

GD is delineated into three clinical variants: the non‐neuropathic type I (GD1), the acute neuropathic type II (GD2), and the sub‐acute neuropathic type III (GD3). Among these, GD2 exhibits the most unfavorable prognosis, manifesting earlier either as a perinatal lethal variant or as the classic type II GD, characterized by pronounced visceral and neurological impairment [2]. The deficiency of the glucocerebrosidase enzyme leads to the accumulation of lipid‐engorged macrophages within the reticuloendothelial system, giving rise to the hallmark clinical manifestations of GD. This accumulation also imparts a distinctive morphological appearance to the affected cells, characterized by an enlarged, granular cytoplasm and eccentrically positioned, rounded nuclei [3]. Skeletal involvement in GD is observed in over 90% of afflicted individuals. The long bones are frequently affected, resulting in pain and diminished mobility [4].

## 2 Case History and Examination

This case report details the clinical presentation, diagnosis, and management of an 8‐year‐old male patient diagnosed with GD, a lysosomal storage disorder. The patient was admitted with a 1‐month history of gradual‐onset abdominal pain, initially mild but progressing to moderate intensity with radiation to the lower abdomen, accompanied by weakness manifesting as difficulty walking. There was no associated fever, drowsiness, vertigo, respiratory distress, vomiting, or altered bowel habits. His past medical history revealed recurrent abdominal distension since 1 year of age, prompting multiple healthcare visits. A bone marrow examination conducted approximately 7 months prior to admission indicated a lysosomal storage disorder, likely GD, corroborated by histopathological findings. Additionally, the patient had undergone a blood transfusion 4 months earlier, receiving two units of packed red blood cells (PRBC) for anemia. His birth history was unremarkable, with a spontaneous vaginal delivery at 3100 g and no perinatal complications. Immunizations were complete per national guidelines, and developmental milestones were age‐appropriate. A family history suggested a possible inherited storage disorder, potentially GD, though no other significant familial conditions were noted. The patient consumed a mixed diet and had no prior surgical history or drug allergies.

Upon admission, physical examination revealed a history of intermittent jaundice starting 5 years earlier, initially ocular and later generalized, alongside progressive generalized weakness and mild abdominal pain. No respiratory or neurological symptoms were reported.

## 3 Differential Diagnosis, Investigations and Treatment

D/D:

1. Niemann‐Pick disease—Another lysosomal storage disorder with overlapping symptoms like hepatosplenomegaly (enlarged liver and spleen) and bone marrow involvement.
2. Leukemia—Certain leukemias, like chronic lymphocytic leukemia, can present with splenomegaly, fatigue, and bone pain, mimicking GD.
3. Multiple myeloma—A cancer of plasma cells that can cause bone pain, anemia, and fatigue, which may resemble skeletal symptoms of GD.
4. Lysosomal acid lipase deficiency (LAL‐D)—A metabolic disorder causing lipid accumulation, leading to hepatomegaly and spleen enlargement, similar to GD.
5. Idiopathic thrombocytopenic purpura (ITP)—A condition with low platelet counts and bleeding tendencies, which might be confused with GD due to bone marrow involvement.

## 4 Investigations

Laboratory investigations showed hemoglobin anemia, thrombocytopenia as shown in Table 1.

**TABLE 1:** Complete blood count of the patient showing thrombocytopenia and anemia.

Test	Result	Unit	Reference range
Total leukocyte count	4100	Cells/cumm	4000–11,000
Neutrophils	42	%	40–75
Lymphocytes	52	%	20–45
Eosinophils	2	%	1–6
Monocytes	4	%	2–10
Basophils	0	%	0–1
Hemoglobin	8.7	g/dL	11–18
Platelets	75,000	Cells/cumm	150,000–400,000
RBC count	3.8	Million/cumm	4.2–5.4
Mean cell volume (MCV)	73	fL	76–96
Mean cell hemoglobin (MCH)	22.6	pg	27–32
Mean cell hemoglobin conc. (MCHC)	31.0	g/dL	32–36
Hematocrit (HCT)	27.9	%	35–54
Red cell distribution width (RDW)	16.8	%	11.5–14

Liver function tests were largely normal. Urine analysis revealed trace proteinuria and numerous white blood cells, while blood culture identified *Staphylococcus aureus* , suggesting a possible infection. The provisional diagnosis was GD with massive splenomegaly, anemia, and stunting, later refined to massive splenomegaly secondary to GD. GD was confirmed via genetic analysis, which showed Type‐1 c.1099C>G variant.

Notable comorbidities included recurrent anemia requiring transfusions.

## 5 Treatment

**FIGURE 1:**
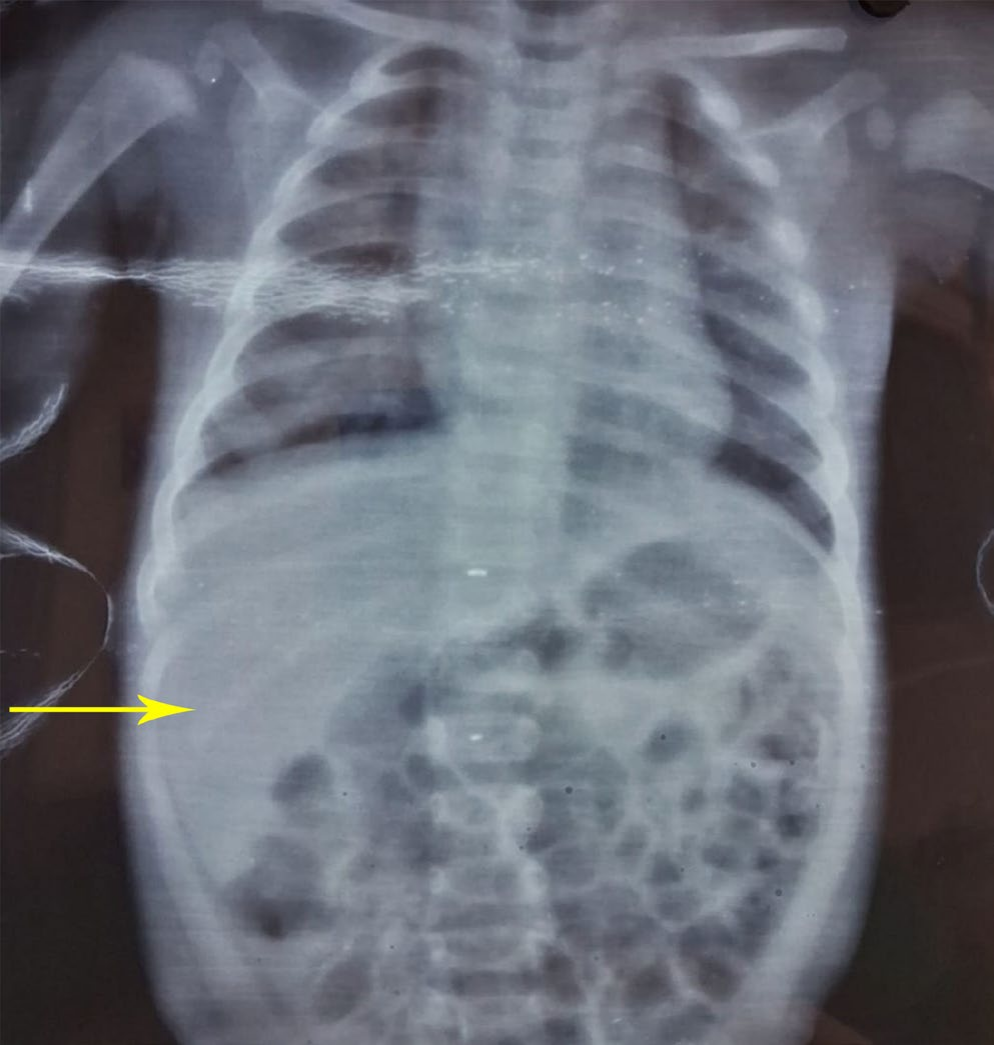
Yellow arrow shows hepatomegaly.

During his two‐day hospital stay in the general ward, the patient received two units of PRBC and symptomatic treatment with Paracetamol as needed. A pediatric surgery consultation recommended elective splenectomy, with follow‐up advised for preoperative immunization. Figure 1 shows hepatomegaly following splenectomy.

The hospital course was uneventful post‐admission fever, and the patient remained hemodynamically stable, improving prior to discharge. Discharge instructions included continued use of Paracetamol as needed and vaccination planning for splenectomy. Consent for the high‐risk procedure was obtained, acknowledging potential complications such as hemorrhage, infection, and anesthesia‐related risks.

In summary, this case underscores the chronic, multisystem nature of GD, with prominent skeletal and visceral involvement, including massive splenomegaly and anemia. The patient's management highlights the importance of supportive care, transfusion therapy, and planned surgical intervention to mitigate disease burden, with ongoing follow‐up critical for optimizing outcomes in this pediatric population.

## 6 Conclusion and Results

GD is a complex lysosomal storage disorder with diverse clinical phenotypes, ranging from the non‐neuropathic type I to the severe neuropathic types II and III, driven predominantly by GBA1 gene mutations and, less commonly, PSAP variants. This case of an 8‐year‐old male with GD type 1 underscores the disease's multisystem impact, manifesting as massive splenomegaly, anemia, and skeletal involvement, consistent with its hallmark features of organomegaly, hematological abnormalities, and bone pathology. The patient's management, reliant on splenectomy due to limited access to enzyme replacement therapy (ERT) or substrate reduction therapy (SRT) in a resource‐constrained setting, highlights disparities in treatment availability and the critical role of supportive care. While ERT and SRT demonstrate efficacy in ameliorating hematological and visceral symptoms, their inability to address neurological manifestations, alongside the persistence of irreversible sequelae such as fibrosis and avascular necrosis, underscores the therapeutic challenges in GD. The variability in disease progression across age groups and subtypes, coupled with the need for rigorous monitoring—particularly in pediatric and GD3 cases—emphasizes the necessity for tailored, multidisciplinary approaches. This case contributes to the broader understanding of GD management, advocating for enhanced access to advanced therapies and comprehensive follow‐up to optimize outcomes in affected populations.

## 7 Discussion

GD manifests across all age demographics, exhibiting a broad array of clinical characteristics. In adult populations, the symptomatic presentation of GD typically emerges prior to the age of 20 years [5]. Nevertheless, the spectrum of this disorder in adults is generally observed to be less severe or to progress more gradually in comparison with its manifestations in pediatric patients.

GD type 1 commonly presents with organomegaly, skeletal abnormalities stemming from bone disease (including radiologically detectable anomalies), and hematological irregularities due to bone marrow involvement. In contrast, neurological manifestations such as parkinsonism or severe outcomes like hydrops fetalis are less frequently observed in type 1 and are more characteristic of types 2 and 3. Consequently, type 1 is distinguished from the other variants primarily by the absence of neurological involvement [5]. However, recent literature has documented occasional neurological features in type 1, notably Parkinson's disease and peripheral neuropathies. In juvenile subacute neurological GD type 3, well‐established manifestations include ocular motor impairment, progressive myoclonic epilepsy, cerebellar ataxia, spasticity, and dementia. Conversely, pediatric GD type 2 is predominantly associated with opisthotonus, bulbar palsy, trismus, psychomotor retardation, and hypertonia, reflecting its distinct and severe neurological profile [6].

**FIGURE 2:**
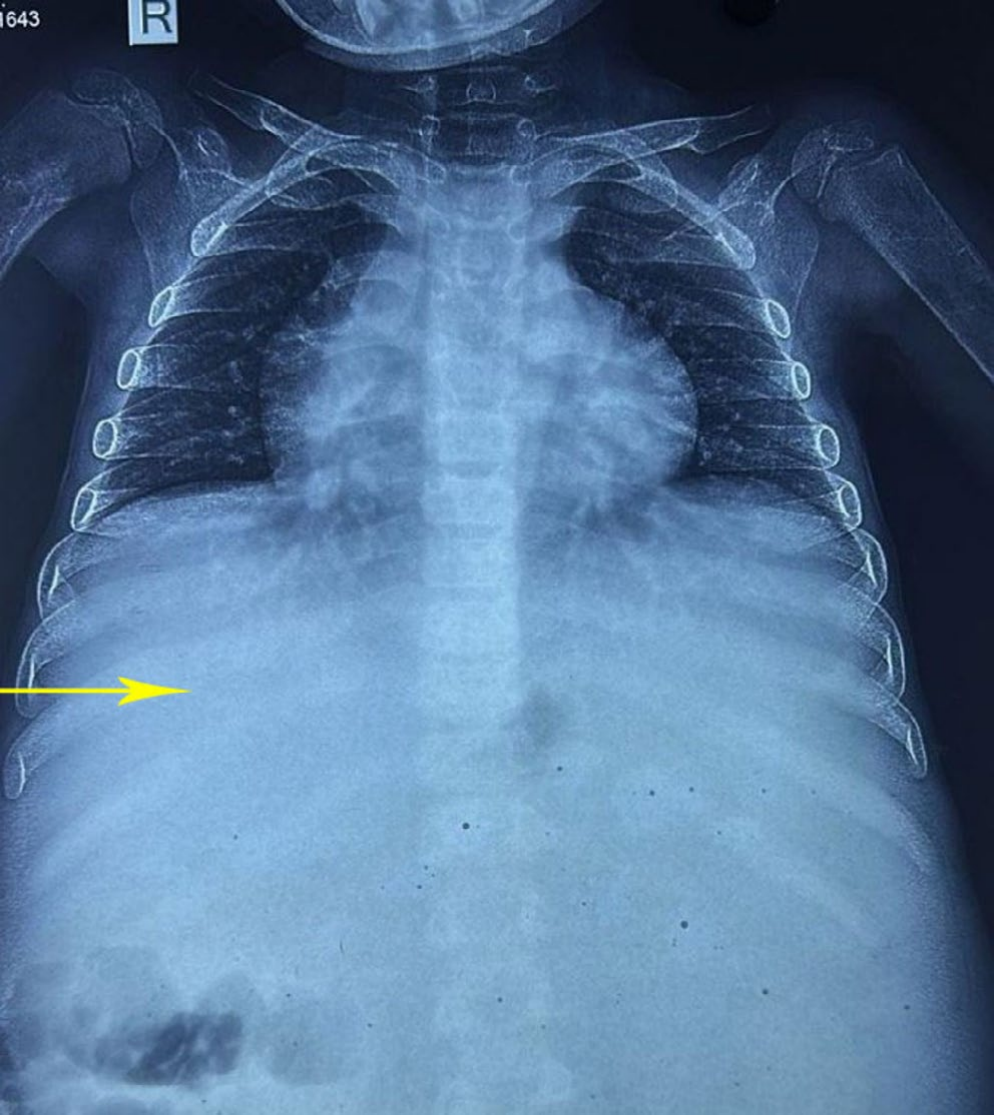
Chest X‐ray prior to splenectomy.

Splenomegaly is documented in over 90% of individuals with GD, while hepatomegaly is observed in 60%–80% of affected patients [6]. Persistent hepatic enlargement, occurring with or without alterations in liver enzyme levels, fibrosis, cirrhosis, and portal hypertension, arises as a result of the intrahepatic accumulation of Gaucher cells and the ensuing secondary inflammatory response. In GD, portal hypertension does not solely result from liver cirrhosis; it may also stem from increased portal system pressure due to splenomegaly or extensive infiltration of Gaucher cells within the liver parenchyma, particularly in patients who have undergone splenectomy. Figure 2 shows chest X‐ray prior to Splenectomy for comparison.

Skeletal involvement represents a frequent complication in GD, with a prevalence ranging from 76% to 94% among patients with GD type 1 [7]. The skeletal manifestations encompass bone mass deterioration and bone marrow infiltration. Radiographic evidence reveals characteristic features such as the Erlenmeyer flask deformity, osteopenia, osteosclerosis, osteonecrosis, and fractures, predominantly affecting the pelvis and lower extremities, with less frequent involvement of the upper limbs.

At present, the therapeutic approaches for GD encompass two primary modalities: ERT and SRT. In ERT, the deficient enzyme is exogenously administered to affected cells, whereas SRT involves the provision of a substrate to curtail the buildup of deleterious substances within cells, thereby mitigating the initiation of a pathogenic cascade. Prior research indicates that intravenous ERT ameliorates osteopenia, bone marrow infiltration, and bone pain. Nonetheless, neither of these interventions demonstrates efficacy in alleviating the neurological manifestations associated with GD [6].

The management of patients with GD requires consistent monitoring through clinical examinations, laboratory tests, and imaging studies. ERT effectively improves blood‐related abnormalities and enhances quality of life within a few months [8]. Key biological markers, such as chitotriosidase, CCL18, and ferritin, show a relatively rapid decrease after starting ERT, often before platelet and hemoglobin levels return to normal [9]. Reduction in the size of the liver and spleen occurs more slowly, typically taking about 2 years. Bone‐related issues tend to improve after 2–4 years of treatment, though some damage—like liver or spleen scarring, avascular necrosis (AVN), or lasting effects of bone infarction—remains permanent [10]. While many patients experience notable progress, full recovery from low blood cell counts or enlarged organs is often not achieved [10]. For those with Gaucher disease type 3 (GD3), additional monitoring of neurological symptoms is essential due to their unique disease characteristics.

In children, monitoring is more frequent and rigorous. A detailed clinical check‐up and a complete set of lab tests are required every 6 months, with imaging used as needed depending on how the disease progresses. This structured approach ensures a comprehensive understanding of the patient's condition over time.

Several study of literature reveals different cases of GD with unique presentation.

A 2‐month‐old male with jaundice, hepatosplenomegaly, and ichthyosis had elevated B12 and holo transcobalamin levels, which were attributed to a severe form of GD and a biomarker of type II GD. The patient, however, expired on the 76th day of life. This gives insight into the severity of cases involving raised B12 levels [11]. A 35‐year‐old man with hepatosplenomegaly, ascites, myelofibrosis, and MPGN was discovered to be a case of GD with genetic analysis, and his symptoms improved following prednisolone and Mycophenolate Mofetil [12]. A 24‐year‐old primipara with idiopathic thrombocytopenia delivered a female infant of 2677 g at 39 weeks of gestation. The neonate developed convulsions, respiratory depression, systemic purpura, and exfoliation of the epidermis. The infant was diagnosed with GD at 37 days of age and expired at 82 days of age. Subsequently, the parents were diagnosed as carriers [1]. A 4‐day‐old female with hyperbilirubinemia and hepatosplenomegaly was diagnosed with GD via genetic analysis. She received ERT, but symptoms worsened, and liver transplantation was performed at 7 months of age; however, the patient expired at 8 months of age [13]. An 11‐month‐old female patient with type 2 GD had her motor activity improved under the treatment of Imiglucerase and ambroxol. The patient's symptoms improved during follow‐up and showed development of 16 months at 3 years and 9 months chronological age and is able to walk independently. This highlights the importance of an early start of ambroxol for the treatment [14].

Our case was treated with splenectomy as there was persistent thrombocytopenia and lack of the facility for ERT or SRT therapy being a resource‐limited setting. Splenectomy prevented the risk of bleeding though it is not the definitive therapy. This case is important for describing the management of GD in a resource‐limited setting.

## Author Contributions

**Bipesh Kumar Shah:** conceptualization, data curation, formal analysis, supervision, validation, visualization, writing – original draft, writing – review and editing. **Diwakar Koirala:** conceptualization, formal analysis, investigation, software, validation, visualization, writing – original draft, writing – review and editing. **Bivek Mishra:** validation, visualization, writing – original draft, writing – review and editing. **Ramesh Sapkota:** validation, visualization, writing – original draft, writing – review and editing. **Manjil Basnet:** validation, visualization, writing – original draft, writing – review and editing.

## Ethics Statement

Ethical Approval was taken for the Submission of Case Report.

## Consent

Written informed consent was obtained from the patient's parents for publication and any accompanying images. A copy of the written consent is available for review by the Editor‐in‐Chief of this journal on request.

## Data Availability

The data that support the findings of this study are available from the corresponding author, Dr. Diwakar Koirala, upon reasonable request.
